# Alpha-1 Antitrypsin Deficiency: Home Therapy

**DOI:** 10.3389/fphar.2021.575402

**Published:** 2021-04-15

**Authors:** Anna Annunziata, Maurizia Lanza, Antonietta Coppola, Paolo Andreozzi, Sara Spinelli, Giuseppe Fiorentino

**Affiliations:** ^1^ Department of Respiratory Pathophysiology Monaldi Hospital, Naples, Italy; ^2^ Gastroenterology Unit, Marcianise Hospital, Caserta, Italy

**Keywords:** alpha-1 antitrypsin (A1AT), home therapy, augmentation therapy, QOL score, SARS, CoV-2

## Abstract

While available in only a few countries, home therapy is a possible strategy for the treatment of alpha-1 antitrypsin deficiency. We want to describe our experience in the management of human alpha-1 antitrypsin using home care intravenous augmentation therapy during this emergency period caused by SARS-CoV2 infection. We assessed the safety of the home treatment and the quality of life of patients enrolled in the program.

## Introduction

Over the last few months, the SARS-CoV2 pandemic has had an impact on chronic therapies in patients with rare disease, such as alpha-1 antitrypsin deficiency (AATD). Augmentation therapy with exogenous AAT is the only specific therapy for the lung disease associated with AATD. The biochemical and clinical efficacy of AAT therapy has been well established in numerous studies–a dose of 60 mg/kg per week (Chapman et al., 2009; McElvaney et al., 2017).

Unfortunately, during the period of emergency, some patients had difficulties accessing the hospital structure for chronic therapies. This was because hospitals were limiting access due to pandemic reorganization and because patients with lung diseases are fragile and would have a potentially high risk for contracting a severe SARS-CoV2 infection.

A recent study described that withdrawal of therapy in 19 compromised AATD patients was associated with poor health outcomes as demonstrated by an increased mean (±standard error) number of exacerbations per patient (1.5 ± 0.2 vs. 0.5 ± 0.1, *p* = 0.002) and an increase in the mean number of hospitalizations in the same patients (0.7 ± 0.1 vs. 0.2 ± 0.1, *p* = 0.003) compared to the same period in the previous year (Gazzettaufficiale, 2012; McElvaney et al., 2020). To avoid the abrupt cessation of augmentation therapy, it has been necessary to activate a home treatment, which is in use in only a few EU countries. In Italy, even though home therapy with alpha-1 antitrypsin has been authorized since 2013 (Gazzettaufficiale, 2012), it is still an unimplemented practice. Home therapy for such patients should be part of the Italian long-term care system. However, due to the system’s complexity, systemic problems regarding the offer of care and eligibility of services can occur owing to the lack of integration between Regions’ and Municipalities’ health policies. AADT augmentation therapy has been demonstrated to be well-tolerated and generally safe. Few and generally mild side effects have been reported and rarely required major interventions or interruption of therapy. Some authors describe side effects typical of intravenous infusion of proteins, including delayed fever resolving spontaneously over 24 h, urticaria, nausea, fatigue and dizziness. Dyspnea, probably related to absolute protein load in the infusion, flu-like symptoms, and, rarely, anaphylactic shock have also been described. No deaths related to AATD augmentation therapy are reported (Wencker et al., 1998; Stoller et al., 2003). Adverse events commonly occur during the first administrations. Initial alpha one antitrypsin administrations are recommended in a hospital setting and in the presence of experienced operators. Currently, there is no literature about the outcomes of safety and quality of life of AATD patients in home treatment.

## Methods

To reduce patients flow to the hospital and prevent human infections, AATD home therapy was activated for 16 patients using a specific patient support program, a home care project, already activated independently throughout Italy. The program allows patients to infuse intravenous augmentation therapy at home by qualified nurses in continuous contact with the referring physician. Fourteen patients in treatment for over one year and two patients with recent treatment initiation were placed in patient support program. The procedures involved in administering home-based intravenous therapy with AATD were the same as in the hospital. An experienced nurse wearing adequate personal protective equipment measured the vital signs, then proceeded to prepare the therapy to be administered. The therapy was administered intravenously according to the schedule. The operator remained at the patient's home throughout the administration. In the end, the vital parameters were checked again and, finally, the venous access removed. The Saint George Respiratory Questionnaire (SGRQ) was not used in all its length. We used a modified Quality of Life (QoL) score for our questionnaire, consisting of six questions (Table 1), to analyze the general state of health, subjective symptoms of the patient and impact of therapy on the patient's and family life. To date, no validated questionnaires are available to assess QoL in patients with AATD. All the individual values were defined based on a 5-point scale: 1) many times a day/very much; 2) every day/much; 3) two or three times a week/not much; 4) once a week/few times; and 5) rarely or never/not at all. There was also a blank space where the patient could leave a comment about their experience of home therapy. The QoL score was calculated as the sum of the points obtained from questions. The minimum possible score was 6, the maximum 30. We compared QoL score for each patient at time zero (during the last hospital administration session) and after 3 months of home therapy.

**TABLE 1:** Questions extracted from SGRQ (St. George Respiratory Questionnaire) to calculate simplified QoL scores.

Please select **ONE** box for each question	
**Question 1.** I cough:	
Many times a day	□1
Every day	□ 2
Two or three times a week	□ 3
Few times	□ 4
Rarely	□ 5
**Question 2.** I bring up phlegm (sputum):	
Many times a day	□ 1
Every day	□ 2
Two or three times a week	□ 3
Few times	□ 4
Rarely	□ 5
**Question 3.** I have shortness of breath:	
Many times a day	□ 1
Every day	□ 2
Two or three times a week	□ 3
Few times	□ 4
Rarely	□ 5
**Question 4.** I have attacks of wheezing:	
Many times a day	□ 1
Every day	□ 2
Two or three times a week	□ 3
Few times	□ 4
Rarely	□ 5
**Question 5.** My therapy (augmentation therapy) interferes with my life?	
very much	□ 1
much	□ 2
not much	□ 3
few times	□ 4
not at all	□ 5
**Question 6.** My respiratory disease is a nuisance to my family, friends or neighbours?	
very much	□ 1
much	□ 2
not much	□ 3
few times	□ 4
not at all	□ 5

Please write any considerations on your current therapy. This section was used to focus on any patient-reported advantages or disadvantages of replacement therapy in hospital and at home.

Patients gave their written informed consent and all data were collected anonymously. A nurse experienced in supporting patients with AATD supervised the completion of the questionnaire. Written comments from patients were also recorded. We surveyed the comments to understand what were the advantages or disadvantages of home treatment perceived by the patient. The observation time was 3 months; however, treatment is continuing at home for all patients. For statistical analysis Student's t-test was used for the continuous variables. All statistical analyzes were performed with SPSS (version 19.0; SPSS Inc. Chicago, IL, United States of America) and *p* < 0.05 was considered significant.

## Results

**FIGURE 1:**
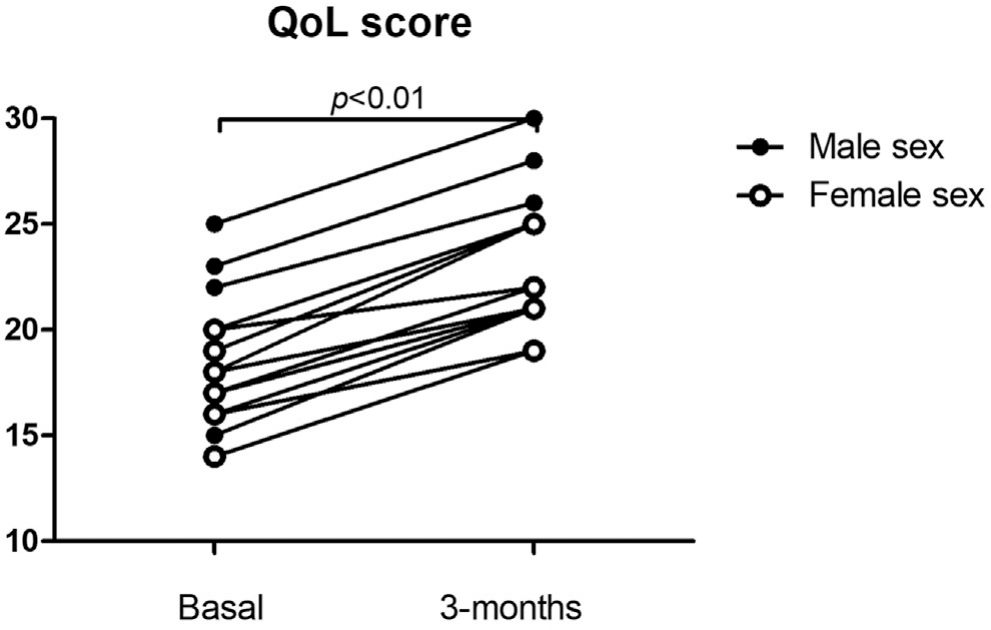
Qql score at time 0 and after 3 months.

Median age (min-max) was 61 years (26–78), there were seven females and nine males. (Table 2) All patients continued home treatment without interruption. The same nurse supported the patient at time zero and after three months, the questionnaire was administered, on both occasions, on the day scheduled for replacement therapy. During the observation time, no adverse events occurred. All the questionnaires completed at 3 months showed an increase in score compared to the questionnaire completed during the last hospital administration session. The quality of life had improved from an index of 18.0 ± 3.0 at the start time (t0) to an index of 22.6 ± 3.3 after 3 months (t3). (Figure 1) The variation was statistically significant (*p* < 0.01). For all patients, the scores of the symptom control questions (“I cough”, “I bring up phlegm”, “I have shortness of breath”, “I have attacks of wheezing”) were unchanged. Instead, all sixteen patients, when asked "my (augmentation) therapy interferes with my life", answered "very much" or "much" at time zero, and “few times” or "not at all" after three months. Also, to the question "my respiratory disease is a nuisance to my family, friends or neighbors?" the same patients responded "very much" or "much" at time zero and "few times" or "not at all" after three months. Seven patients provided written comment. Three explained that the advantage of home therapy was to be independent of a family member or friend who had to accompany them to the hospital. Three others wrote that they could undergo home therapy without losing working or university day. These patients reported being very stressed while going to the hospital to undergo intravenous therapy. This was especially true for patients who lived far away from the hospital. Only one patient reported feeling safer when he was treated at the hospital than at home because he felt he was monitored more carefully.

**TABLE 2:** Feature of patients.

Female sex, *n* (%)	7 (43.75%)
Age, median age (min-max)	61 years (26–78)
Smoking habits	
Active Smokers, *n* (%)	0 (0%)
Former Smokers, *n* (%)	7 (43.75%)
No Smokers, *n* (%)	9 (56.25%)
TC Features	
Panlobular Emphysema, *n* (%)	10 (62.5%)
Centrilobular Emphysema, *n* (%)	3 (18.8%)
Bronchiectasis, *n* (%)	2 (12.5%)
Fibrosis, *n* (%)	1 (6.3%)
Alfa1AT (mg/dl), (mean ± SD)	83.4 ± 23.17
Basal QoL score, (mean ± SD)	3.0 ± 0.51
3-months Follow Up QoL score, (mean ± SD)	3.77 ± 0.54

## Discussion

Augmentation therapy with alpha-1 antitrypsin slows disease progression (Chapman et al., 2015; McElvaney et al., 2017) and discontinuation of treatment was associated with poor health outcomes, increased number of exacerbations and hospitalizations (Alkins and O’Malley, 2000; Sclar et al., 2012; McElvaney et al., 2020). Some studies concluded that augmentation therapy is a cost-effective strategy for managing AATD patients with COPD (Alkins and O’Malley, 2000; Sclar et al., 2012). An observational study among Spanish patients also showed that these patients experienced a significant decrease in hospitalization costs and incidence of exacerbations following the start of augmentation therapy (Barros-Tizón et al., 2012).

Despite the known benefits, some patients had difficulty starting treatment or continuing it. In a recent publication, the most frequently reported practical difficulties with AATD infusions were infusion time, frequency of infusions, overbooked outpatient clinic, and treatment provided by only one center. Many patients also find it difficult to reach the hospital because they reside far away. Clinicians, trying to improve the convenience of AATD patients in therapy, often consider alternative dosing strategies (bi-weekly dosing); reasons for this consideration include coverage of holidays and individuals in full-time employment (Horváth et al., 2019). Self-administration is not viable for all patients and the safety issues surrounding intravenous administration is the main disadvantage. Home therapy is a possible strategy, available in a few countries. It is the only treatment option in Ireland and France; in Poland, regular treatment is provided at home or is available at hospital outpatient clinics (Horváth et al., 2019). During the COVID-19 pandemic, the difficulties in accessing therapy were accentuated by the reduced access to hospital and the need to preserve fragile patients, therefore the patient support program was activated to avoid the interruption of therapy that many patients would have incurred.

All patients adhered to the home care support program and continued treatment without interruption and side effects. The quality of life measured with a short questionnaire showed a positive change after three months of home therapy. The patients reported being less stressed undergoing treatment at home, burdening less on their family and friends, and feeling less interference with their personal and family life. It should also be considered that some patients lose days of work or study to carry out the therapy at the hospital, while at home it can be carried out before or after work/study.

Our results highlight that the home care of AATD patients, who need augmentation therapy, contributes to the optimal care and improvement of the patients’ quality of life. Again, a home-based administration is safe and demonstrates no side effects. According to the results of other studies, home care is also practicable for several years (Wilke et al., 2018). Limitations of the study are the use of a reduced version of the SGRQ currently not validated and the small number of patients. In our study, we took advantage of a free patient support program to allow patients to continue therapy at home, the cost of the specific patient support program were fully bore by Grifols Italy. Consequently, the costs for the public health system were zero. However, due to the little diffusion of home therapy, to date, there are no studies that compare the cost of the hospital with home assistance.

In conclusion, convenience for the patient is viewed as the most important advantage of home treatment. The quality of life of our patients is the main objective, especially in the case of people with rare diseases with progressive deterioration of daily living. Larger studies are needed to evaluate the impact on the quality of life of home therapy, the sustainability of the costs of a home treatment, the advantage in terms of working days saved for the patient and for the caregiver.

## Data Availability

The raw data supporting the conclusions of this article will be made available by the authors, without undue reservation.
